# Emotional Context Shapes the Serial Position Curve

**DOI:** 10.3390/brainsci12050581

**Published:** 2022-04-29

**Authors:** Fabio Giovannelli, Iglis Innocenti, Emiliano Santarnecchi, Elisa Tatti, Stefano F. Cappa, Simone Rossi

**Affiliations:** 1 Section of Psychology—Department of Neuroscience, Psychology, Drug Research and Child’s Health (NEUROFARBA), University of Florence, 50135 Florence, Italy; fabio.giovannelli@unifi.it; 2 Siena Brain Investigation and Neuromodulation Lab (Si-BIN Lab), Unit of Neurology and Clinical Neurophysiology, Department of Medicine, Surgery and Neuroscience, University of Siena, 53100 Siena, Italy; iglisinnocenti@gmail.com; 3 Berenson-Allen Center for Noninvasive Brain Stimulation, Beth Israel Deaconess Medical Center, Harvard Medical School, Boston, MA 02115, USA; esantarn@bidmc.harvard.edu; 4 Department of Cognitive Neurology, Harvard Medical School, Boston, MA 02115, USA; 5 Department of Molecular, Cellular & Biomedical Sciences, CUNY, School of Medicine, New York, NY 10031, USA; etatti@ccny.cuny.edu; 6 Institute for Advanced Study, IUSS, 27100 Pavia, Italy; stefano.cappa@iusspavia.it; 7 IRCCS Mondino Foundation, 27100 Pavia, Italy

**Keywords:** emotional context, memory, serial position curve, primacy, recency

## Abstract

Emotional contexts affect memory processes. However, the impact of contextual priming as a function of the emotional valence on the recall of neutral information is not fully understood. The aim of the present study was to evaluate how a conditioning of emotional context during encoding may influence the subsequent memory of otherwise neutral materials in a well-established phenomenon as the serial position effect. Participants performed a free recall task for neutral words in three conditions: (i) word list alone; (ii) word list coupled with positive or neutral images; and (iii) word list coupled with negative or neutral images. Images were presented before each word stimulus. In three different experiments, the emotional context during the word list presentation was manipulated separately for primacy and recency clusters, and for the middle words (‘middlecy’). Emotional context affects free recall of neutral stimuli, changing the serial position curve effect across conditions. Namely, emotional images presented in the primacy and recency clusters worsen accuracy, whereas their occurrence in the ‘middlecy’ cluster reduces the oblivion. The present findings show that the typical pattern related to the serial position curve for neutral information can be shaped by the conditioning of emotional context. Findings have implications in medical-legal contexts in the case of the recollection of events with high emotional content.

## 1. Introduction

Extensive psychological literature has documented the impact of the emotional quality of an item as well as the emotional state of an individual on the encoding, consolidation, and retrieval of memories. Emotional items are typically better remembered and are more robust against forgetting than neutral items [1,2]. Emotional states can enhance memory for both neutral and emotional items [3,4,5,6]. According to the cue-utilization hypothesis [7], the presence of emotional information narrows the scope of available attention, requiring that we “select” the parts to which we attend. As the most pertinent information is likely to be the emotional information, this leads to preferential processing of the emotional stimulus at the cost of the peripheral, non-emotional information. A number of theories have been proposed to account for this emotional memory trade-off (see, for example, central vs. peripheral details [8,9,10]).

The impact of emotion on memories is not limited to the valence/arousal properties of the encoded stimuli. The literature on emotional context reports that emotional states foster the retrieval of both neutral and emotional items [6]. Concerning the role of the emotional context on information without emotive valence, some behavioral studies have combined emotional stimulation with the encoding of neutral stimuli and highlighted that a positive context enhances the retrieval of neutral stimuli [11]. Neuroimaging studies showed that the free recall of neutral material was enhanced when encoded in a positive context [6], an effect that was not behaviorally evident during a passive recognition task [12]. The effect of emotional context (modulated as positive or negative emotional arousal) on memory was also observed in animal models [13,14]. Therefore, emotional context seems to have a differential effect on the subsequent memory retrieval, depending on its valence.

We reasoned that a suitable and original way to study the impact of different emotional contexts on memory retrieval is to verify whether and how much they influence a solid cognitive phenomenon such as the serial position effect. Since its first description in 1878 [15], it was pointed out that the balance between learning and forgetting is deeply influenced by the serial position of the information that has to be encoded. The U-shaped profile of an immediate recall performance is characterized by two specific effects: the higher recall probability of the items positioned at the beginning (primacy effect) and at the end of a list (recency effect). The primacy effect can be attributed to rehearsal-dependent transfer of the initial items from short-term memory to long-term memory, whereas the recency effect results from the prompt availability of the last encoded items in the short-term store [16,17]. Consequently, items in the middle of a list are disadvantaged and prone to oblivion: too little rehearsed to get into long-term memory and too old to be kept in the short-term store [18].

Given the influence of different emotional contexts on the recall of neutral information, a further step could be to better characterize their effects on well-known memory processes. Therefore, we applied emotional context during the encoding of neutral verbal items by associating each serially presented target-word to a picture that could be neutral or convey a negative or a positive feeling. This allowed us to explore the emotional impact on the serial position effect.

## 2. Materials and Methods

### 2.1. Participants

A total of 67 right-handed healthy volunteers (39 women; mean age: 29.1 years, range: 23–35) with no history of neurological and psychiatric disease or drug abuse, normal hearing, and normal or corrected-to-normal vision were included in the study. Participants were mainly recruited from the student community of the University of Siena (School of Medicine, medical and psychology post-degree specialization schools, and PhD courses).

All participants gave their written informed consent to the procedure. All data were collected and processed anonymously. This study, conducted according to the guidelines of the Declaration of Helsinki, is part of a large set of behavioral and non-invasive studies on memory processing and other cognitive functions approved by the Local Ethical Committee of the University of Siena (IRB Protocol: Apollo). Prior to the evaluation, each subject was blind to the purpose of the study, which was carefully explained after the completion of the evaluation.

### 2.2. Stimuli

Visual stimuli consisted of a set of 150 images selected from the Geneva Affective Picture Database (GAPED; [19]). Based on the normative ratings of emotional valence and level of arousal provided by Dan-Glauser and Scherer [19], 25 images were classified as ‘positive’ (pictures representing human babies, young animals, and natural landscapes), 25 images as ‘negative’ (pictures representing human concerns), and 100 as ‘neutral’ (pictures representing objects, buildings, and furniture) (Table A1).

Fifteen lists of 15 unrelated Italian medium-high frequency words (according to the Corpus and Frequency Lexicon of Written Italian CoLFIS, (http://www.istc.cnr.it/material/database/colfis/, accessed on 25 April 2022), ranging from 4 to 12 letters in length (two or three syllables) were prepared (Table A2). Stimuli consisted of digital audio-recordings of 225 words spoken by a male speaker. This set of stimuli was created for a previous experiment in our laboratory [20]. Each stimulus word consisted of a 1 s epoch saved in a digital sound file with a sampling rate of 44 kHz (16-bit, stereo, WAV-format). Auditory stimuli were normalized by −10 dB root mean square using the software Audacity to ensure similar loudness. In order to minimize click noise at sound onset and offset, 10 ms rise and fall times were applied to all sounds.

### 2.3. Experimental Protocol

The study consisted of three main experiments and one control experiment in which the same stimuli were employed. The participants were randomly divided into four groups and each subject took part in only one experiment.

#### 2.3.1. Main Experiments

**Figure 1:**
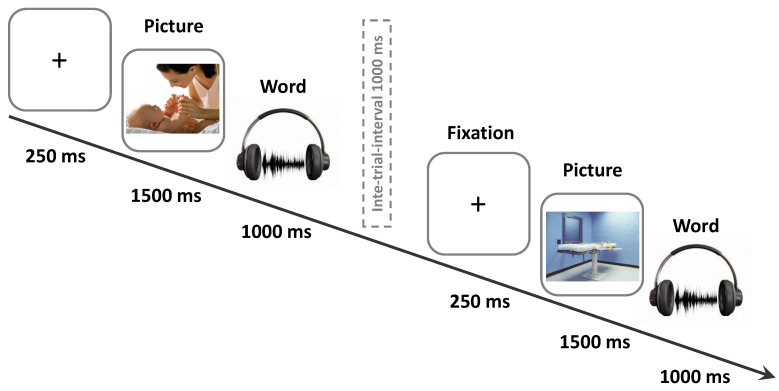
Encoding phase of the memory task: 15 words were presented acoustically; pictures were displayed for 1500 ms immediately before the sound-word presentation.

All participants performed a free recall memory task, of which they were informed. They were seated in front of a monitor, wearing headphones connected to a personal computer. Words were presented acoustically, at a comfortable sound level via headphones, along three experimental conditions: (i) word list alone (control); (ii) word list with positive or neutral images (positive context); and (iii) word list with negative or neutral images (negative context). In each experimental condition, five 15-word lists were administered. In the conditions in which word lists were presented with images, five blocks of images were used (each block of images consisted of five emotional modulators and 10 neutral images; for a total of 75 trials). All pictures were displayed for 1500 ms immediately before the sound-word presentation (Figure 1). In the word list alone condition, a blank picture was presented for 1500 ms (Figure 1). The inter-trial interval was 1000 ms. Immediately after the end of the presentation of each list, subjects were requested to recall as many words as they could. Software E-prime 1.2 (Psychology Software Tools, Inc., Sharpsburg, PA, USA) was used for stimuli presentation.

**Figure 2:**
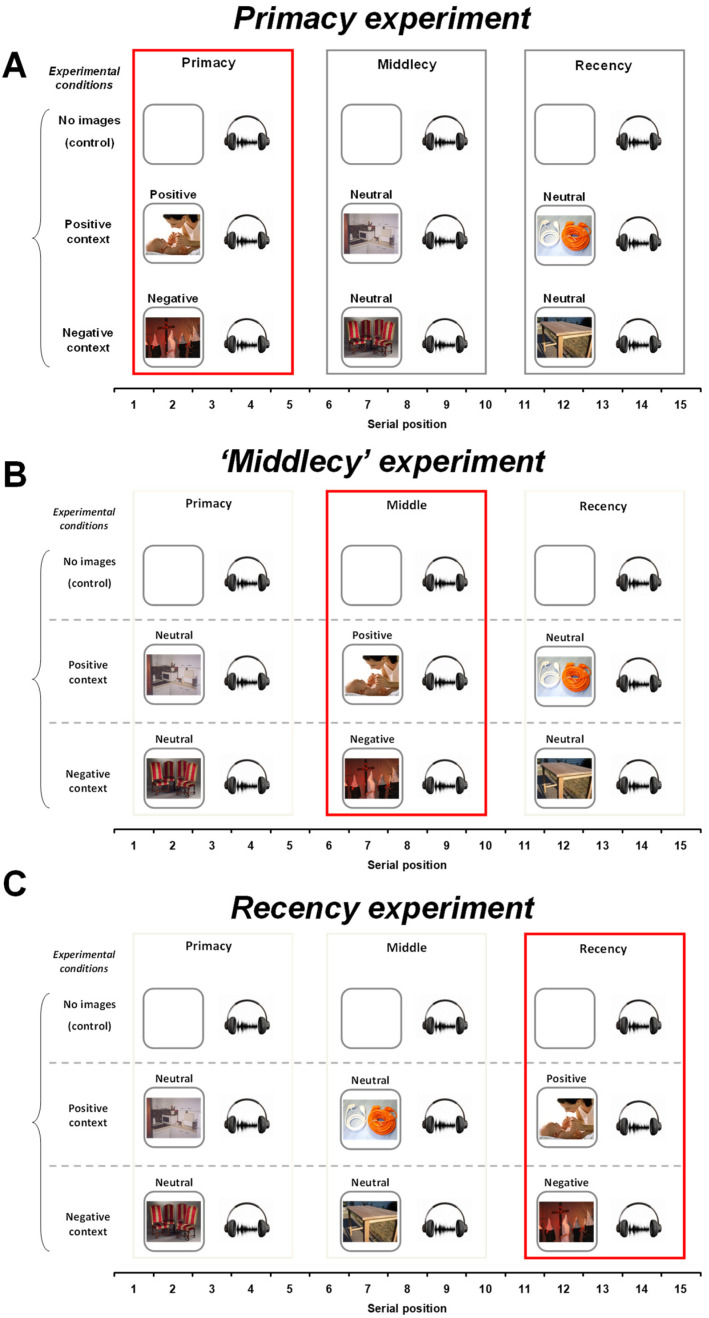
Schematic drawing representing the experiments. (**A**) In the ‘primacy experiment’, words of the primacy cluster was preceded by positive or negative images whereas words of the middle or recency clusters were preceded by a neutral picture. (**B**) In the ‘middlecy experiment’, emotional context (positive or negative) was manipulated only for words of the middle cluster. (**C**) In the ‘recency experiment’, emotional context was manipulated only for words of the recency cluster. Each experiment included a control condition with words presented alone. In the conditions in which word lists were presented with images, five blocks of images were used (each block of images consisted of five emotional modulators and 10 neutral images; for a total of 75 trials).

Each 15-word list was grouped into three clusters: primacy cluster (serial positions from 1 to 5), middle cluster (from here thereafter ‘middlecy’, 6–10), and recency cluster (11–15). In each experiment, the emotional context during the word list presentation was manipulated separately for primacy, middlecy, and recency clusters (Figure 2).

For the primacy experiment (*n* = 18 subjects; 10 women; mean age: 28.6 years, range: 24–35), in the blocks in which word lists were presented with images, each word of the primacy cluster was preceded by positive or negative images whereas words of the middlecy or recency clusters were preceded by a neutral picture (Figure 2A).

For the middlecy experiment (*n* = 20 subjects; 12 women; mean age: 29.2 years, range: 23–35), emotional context (positive or negative) was manipulated only for words of the middlecy, whereas words of the primacy or recency clusters were preceded by a neutral picture (Figure 2B).

Finally, for the recency experiment (*n* = 18 subjects; 11 women; mean age: 29.4 years, range: 23–35), emotional context was manipulated only for words of the recency cluster (Figure 2C).

All other protocol details were identical for all experiments. In each experiment, the order of conditions and coupling between blocks of stimuli and experimental conditions were randomized and counterbalanced across subjects. In each block and list, visual pictures were matched for valence and arousal rates (Table A1) according to the normative data [19].

#### 2.3.2. Control Experiment

In order to rule out the effect of the mere presence of the images during the memory task, a control experiment (*n* = 11 subjects; 6 women; mean age: 29.5 years, range: 24–34) was performed. Namely, words were presented in two experimental conditions: (i) word list alone (no images); and (ii) words with neutral images (i.e., each word was preceded by an emotionally neutral picture).

### 2.4. Data Analysis

Two experimenters independently took note of the remembered words and of their serial position in the presented list. Any occasional intrusion (i.e., words incorrectly rated as listened by subjects) was also noted. However, since they occurred rarely (less than 5%), they were not reported.

Data from the three main experiments (primacy, middlecy, and recency) were separately analyzed.

For each participant, behavioral performances were separately evaluated by each list and experimental condition through accuracy measures (number of recalled items). The dependent variable was the percentage of words correctly recalled as a function of word presentation order. The mean percentage was calculated for each cluster. The collected data were entered into a two-way repeated-measures ANOVA with ‘emotional context’ (three levels: positive, negative, no images) and ‘serial position cluster’ [three levels: primacy (1–5), middlecy (6–10), recency (11–15)] as within-subject factors.

For the control experiment, data were analyzed by a two-way repeated-measures ANOVA with ‘experimental condition’ (two levels: no images vs. neutral images) and ‘serial position cluster’ [three levels: primacy (1–5), middlecy (6–10), recency (11–15)] as within-subject factors.

To correct violations of the sphericity assumption, Greenhouse–Geisser corrections were applied when necessary. Post-hoc tests were performed by the Bonferroni test. For all analyses, significance was set at *p* < 0.05. In addition, partial eta squared (η_p_^2^) was calculated as the effect size.

## 3. Results

Specific details about the modulation observed during the primacy, middlecy, and recency experiments are reported below.

### 3.1. Primacy Experiment

**Figure 3:**
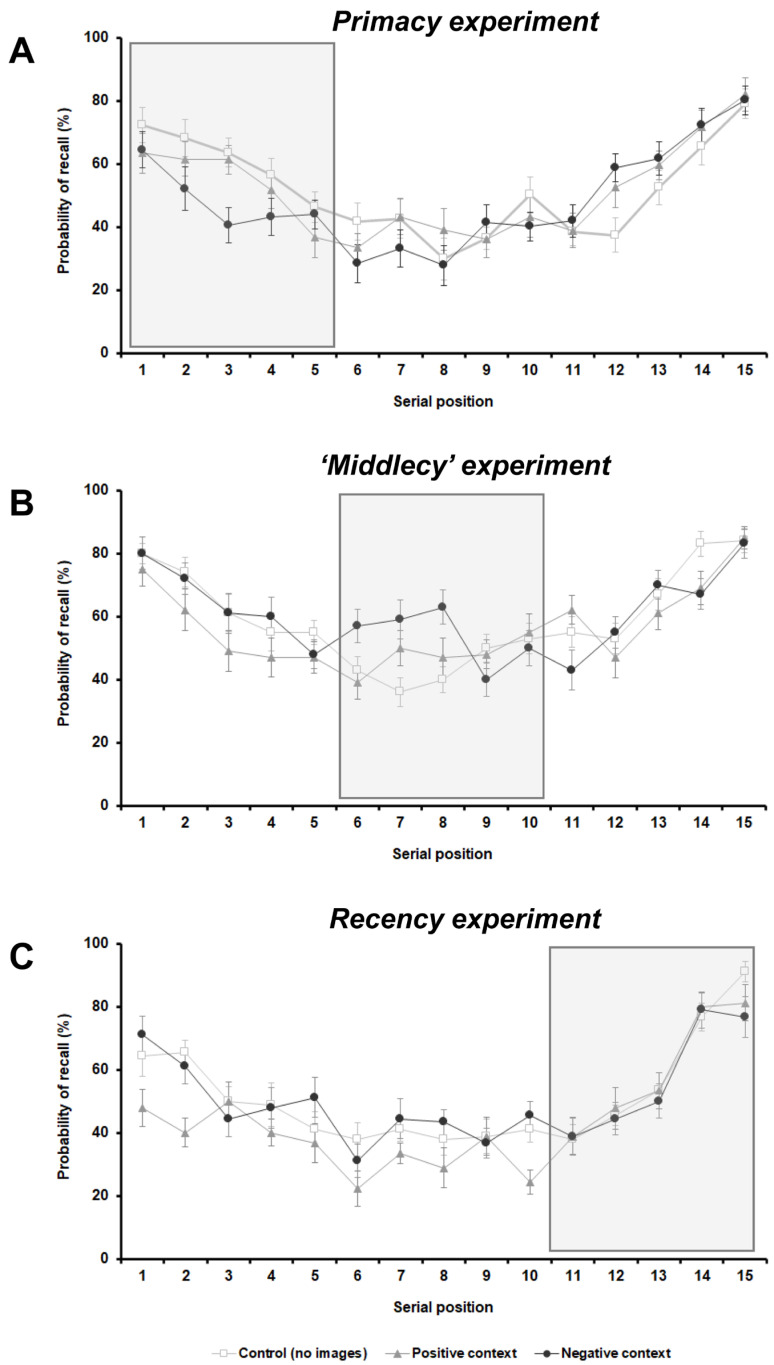
The ‘U-shaped serial position curve’ of the 15-word list during the immediate free recall task in the primacy (**A**), ‘middlecy’ (**B**), and recency (**C**) experiments.

**Figure 4:**
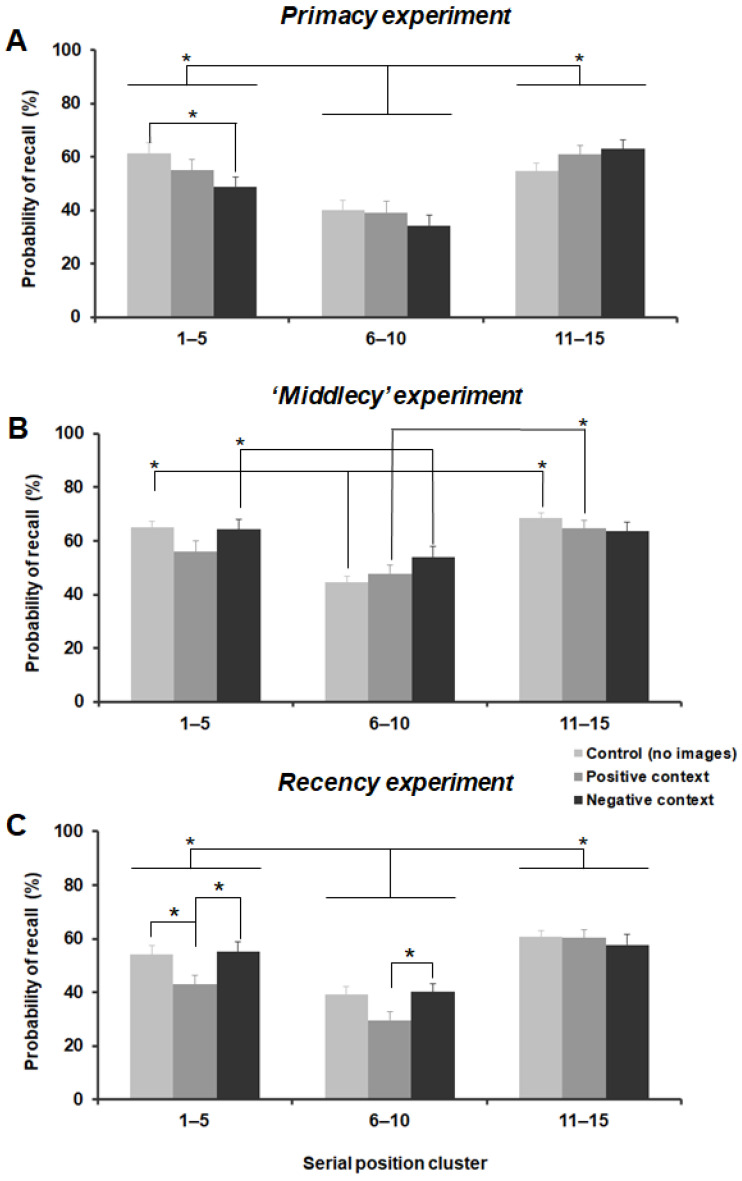
Percentage of correct responses expressed as clusters, according to each experimental condition in the primacy (**A**), ‘middlecy’ (**B**), and recency (**C**) experiments. * *p* < 0.05.

In the primacy experiment, the two-way repeated-measures ANOVA showed that the main effect of ‘emotional context’ was not significant (F_2,34_ = 1.340, *p* = 0.275, η_p_^2^ = 0.073), whereas the main effect of ‘serial position cluster’ was significant (F_2,34_ = 27.762, *p* < 0.001, η_p_^2^ = 0.620). Notably, the interaction between these two factors was also significant (F_4,68_ = 7.402; *p* < 0.001, η_p_^2^ = 0.303). As expected, participants were more accurate in recalling words presented at the beginning and at the end of the list (primacy and recency effects; Figure 3A and Figure 4A). Post-hoc comparisons (Bonferroni corrected) confirmed that, for all conditions (no images, positive, and negative), the percentage of recalled words was significantly higher for clusters 1–5 (primacy) and 11–15 (recency) compared to the words in the middle of the curve (no images: *p* < 0.001 and *p* = 0.012; positive: *p* < 0.001 for both comparisons; and negative: *p* = 0.003 and *p* < 0.001).

Moreover, post-hoc comparisons revealed that the percentage of recalled words in cluster 1–5 (primacy) was significantly lower for words presented after pictures with negative valence (negative emotional context condition) compared to the control (no images) condition (*p* < 0.001) (Figure 3A and Figure 4A). Results of this experiment suggest that a negative context had a detrimental effect on the free recall for items in the primacy position.

### 3.2. Middlecy Experiment

Similarly, in the middlecy experiment, the main effect of ‘emotional context’ was not significant (F_2,38_ = 2.352; *p* = 0.109, η_p_^2^ = 0.110) whereas the main effect of ‘serial position cluster’ was significant (F_2,38_ = 18.016; *p* < 0.001, η_p_^2^ = 0.487). Again, the interaction between these two factors was significant (F_4,76_ = 3.130; *p* = 0.019, η_p_^2^ = 0.141).

The typical primacy and recency effects emerged in the control (no images) condition, whereas in the positive and negative conditions, the ‘oblivion’ effect in the middle cluster was less evident (Figure 3B and Figure 4B). Namely, post-hoc comparisons (Bonferroni corrected) revealed that for the no images condition, the percentage of recalled words was significantly higher for clusters 1–5 (primacy) and 11–15 (recency) compared to the words in the middle of the curve (*p* < 0.001 for both comparisons). For the positive condition, only the recency effect was observed: the percentage of recalled words was significantly higher for cluster 11–15 (recency) compared to cluster 6–10 (middle) (*p* = 0.002). For the negative condition, only the primacy effect was seen, and the percentage of recalled words was significantly higher for cluster 1–5 (primacy) compared to the 6–10 (middle) cluster (*p* = 0.019).

Overall, emotional context (particularly negative context) enhanced the recall of un-related words in the middle cluster position (Figure 3B and Figure 4B).

### 3.3. Recency Experiment

The two-way repeated-measures ANOVA showed that the main effects ‘emotional context’ and ‘serial position cluster’ were both significant (F_2,34_ = 5.360; *p* = 0.018, η_p_^2^ = 0.240, and F_2,34_ = 33.794; *p* < 0.001, η_p_^2^ = 0.665, respectively). Additionally, the interaction between these two factors was significant (F_4,68_ = 2.673; *p* = 0.039, η_p_^2^ = 0.136).

Similarly to the primacy experiment, participants were more accurate in recalling words presented at the beginning and at the end of the list (primacy and recency effects; Figure 3C and Figure 4C). Post-hoc comparisons (Bonferroni corrected) confirmed that, for all conditions (no images, positive, and negative), the percentage of recalled words was significantly higher for clusters 1–5 (primacy) and 11–15 (recency) compared to the words in the middle of the curve (no images: *p* = 0.002 and *p* < 0.001; positive: *p* = 0.018 and *p* < 0.001; and negative: *p* = 0.005 and *p* < 0.001).

Moreover, post-hoc comparisons revealed that the percentage of remembered words in primacy was significantly lower for words associated with positive images versus either no image and negative conditions (*p* = 0.006 and *p* = 0.041, respectively) (Figure 3C and Figure 4C). In the middlecy cluster, the percentage of recalled words was significantly lower for the positive context versus negative image condition (*p* = 0.022). Results of this experiment suggest that a positive context in the recency cluster had a retroactive detrimental effect on the free recall for items in the primacy position and partly in the middle position.

### 3.4. Control Experiment

**Figure 5:**
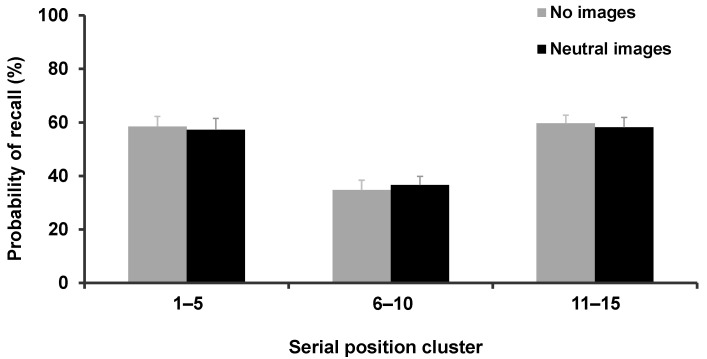
Percentage of correct responses expressed as clusters, according to each experimental condition in the control experiment.

In the control experiment, the main effect of ‘serial position cluster’ was significant (F_2,20_ = 18.868; *p* < 0.001, η_p_^2^ = 0.652) whereas the main effect ‘experimental condition’ (F_1,10_ = 0.736; *p* = 0.444, η_p_^2^ = 0.069) and the interaction between these two factors (F_2,20_ = 2.352; *p* = 0.109, η_p_^2^ = 0.110) was not significant (No images: 59.3 ± 15.6; 35.6 ± 13.9; and 61.1 ± 11.2 for primacy, middlecy, and recency clusters, respectively; neutral images 53.5 ± 14.7; 36.0 ± 12.5; 60.4 ± 14.4 for primacy, middlecy, and recency clusters, respectively) (Figure 5).

## 4. Discussion

The aim of the present study was to evaluate how the conditioning of emotional context presented during the encoding may influence the subsequent memory of neutral materials. Emotional context affects the free recall of neutral stimuli, modulating the serial position curve effect (a solid neuropsychological protocol) across experimental conditions. Emotional images presented in the primacy and recency clusters of the word list worsened recall accuracy compared to the control condition, whereas their occurrence in the middle of the list increased retrieval accuracy: the emotional context reduced the oblivion of the words presented in the middle of the list, what we refer to as ‘middlecy’. This effect emerged for the items presented in the cluster corresponding to the emotional context manipulation, except for the recency condition, in which some retroactive effects were seen. These effects were not due to the mere presence of the images during the memory task as in a control experiment in which each word was preceded by a neutral picture, the memory performance was similar to the recall of the word list alone.

One of the primary ways emotion alters memory encoding is through influencing attention and perception, thus changing what information comes into memory. According to the arousal-biased competition theory recently proposed by Mather and Sutherland [21], emotional arousal may amplify the effects of stimulus salience in attention and memory, resulting either in the enhancement or impairment of memory, depending on the goal-relevance and priority of the neutral information to be memorized.

The effect that emerged in the primacy and recency manipulation experiments of our study is partly in keeping with recent evidence that, if an emotional context is presented before a neutral target stimulus, the attentional resources to process the following items are reduced, resulting in an impairment of recognition rate [22,23]. An unexpected result of the present study is that an emotional context, particularly if negative, can contrast the physiological oblivion to which middle items of a word list are subjected, as revealed by the memory enhancement observed in the ‘middlecy’ manipulation condition. This opposite effect suggests the existence of a complex relationship between the cognitive processes involved in the serial position effect and the arousing response to an emotional context. A possible interpretation is that the emotional context could either interfere with or boost the allocation of attentional resources [24]. A model of serial position effects hypothesized an attentional primacy gradient in serial recall memory tasks [25], with maximal attention resources devoted to the early stimuli during encoding. Evidence of this attentional gradient for initial items also emerged in the free recall tasks as reported by several electrophysiological studies [26,27,28]. As a consequence, in the primacy manipulation condition, the emotional context would interfere with the transfer to long-term memory by capturing attentional resources that are less available for successful encoding of the initial list items. In contrast, in the ‘middlecy’ experiment, the presence of emotional stimuli would increase the depth of processing in the middle part of the serial curve, in which less competition for attentional resources occurs, also prioritizing neutral information.

In the recency experiment, the detrimental effect on the subsequent memory may depend on interference with short-term memory processes involved in the retrieval of these items [23]. This hypothesis is in keeping with models showing that the availability of working memory resources for maintaining stimulus-processing priorities is crucial for directing attention to relevant information, minimizing the intrusion of irrelevant distractors [29].

Therefore, we may speculate that emotional contextual stimuli in the first and last clusters of the serial position may selectively impair the long-term and short-term storage responsible for the primacy and recency effect, respectively, while conversely boosting items in the middle cluster.

Two other hypotheses, not necessarily mutually exclusive, can be advanced to account for the effect observed with the items in the middle position of the curve. First, the emotional modulators could have reduced interference among the items (i.e., interference from preceding and subsequent words). Levens and Phelps [30] used a recency-probes paradigm to investigate how emotional stimuli affected the interference resolution. They found that arousing emotional stimuli produced less proactive interference compared to neutral stimuli, as revealed by faster reaction times during the recognition memory task. The authors suggested a facilitation of interference resolution by highly arousing emotional content [30]. Similarly, we speculate that emotional context may facilitate the recall of neutral words in the middle position of the curve, usually more sensitive to the effect of interference. Second, an isolation or “von Restorff effect” [31] cannot be ruled out. Items in the middle of a list that differ from the others for one dimension are more likely to be remembered in a free recall task. In our study, emotional context mediated by the pictures proceeding each word can be considered as a distinctive feature that facilitate the subsequent recall. However, a previous event-related potential study [32] evaluated the von Restorff effect, manipulating the distinctiveness of an item in the middle position of a words list by a physical attribute (color font) or by the emotional context (highly arousing background picture). They found that only words isolated by color, but not by arousing background, were better recalled [32]. Some differences in the experimental procedures may explain these contrasting results. Namely, we manipulated the middle cluster (i.e., five words) instead of a single item; the emotional images preceded the word presentation so that stimuli were not concomitant; and the words were presented acoustically and not in the same modality of the emotional priming. Further studies are needed to better understand the contribution of these phenomena (interference and von Restorff effect) on the relationship between emotional context and memory processes.

The effects of emotions on memory are likely to be mediated by subcortical limbic circuits altering hippocampal-dependent memories through their influence on perception and attention. In particular, investigations of the brain systems mediating the influence of emotions on attention and perception highlight a role for the amygdala, the key structure signaling the presence of emotional and relevant stimuli in the environment [33,34,35].

### Limitations of the Study

Some limitations in our design must be acknowledged and considered for future investigations. First, the impact of emotional context on primacy, ‘middlecy’, and recency clusters was evaluated in different independent matched groups along three separate experiments. It will be useful to conduct a further study using a within-subjects design to confirm and extend the findings that emerged in the present study. A number of models have been proposed, beyond the dual-store model of memory, to account for free recall dynamics such as the temporal context model (TCM) [36,37], and the context maintenance and retrieval model (CMR) [38]. These models emphasize the interactions between items and context during both encoding and retrieval. Namely, according to the TCM, during the encoding, phase items are associated with a gradually-changing mental context representation [37]. Then, during retrieval, item-to-context associations allow mental representation to favor memory recall. The CMR model expands the TCM by including the influence of non-temporal features (i.e., semantic similarity between items and source context) on the recall process [38]. Recently, Talmi et al. [39] proposed an extension of the CMR model (the emotional CMR) to account for the emotional memory enhancement effect. In the present study, we reported a first characterization of the emotional context effect (using images as modulators) on the serial position curve by specifically manipulating primacy, ‘middlecy’, and recency clusters. Therefore, further investigations are required to explore how the valence of emotional context can impact the dynamics of free recall for neutral stimuli in the framework of current models of episodic memory. Moreover, ad-hoc experiments are needed to disambiguate the relative contribution of valence and the arousal of the emotional context modulators in shaping free recall. Finally, it will be interesting to evaluate, in future studies, the influence of individual factors (e.g., level of clinical and subclinical mood disorders such as depression) in mediating the relationship between emotional context and the processes underlying the serial position curve.

## 5. Conclusions

Understanding the influence of emotional contexts, particularly negative, on mechanisms involved in memory retrieval could have potential clinical implications for psychiatric disorders characterized by memory dysfunction such as post-traumatic stress disorder (PTSD), mainly if the negative memories are associated with autobiographical signatures [40] as well as in medical–legal contexts, when exact recollection of stressful, generally autobiographical, events is required.

In conclusion, our study demonstrated that the serial position curve, a solid neuropsychological construct resistant to external manipulations (especially in its central items) including brain stimulation protocols [20], is modifiable by coupling pictures with emotional valence to the neutral words to be remembered. Such an emotionally-driven reduction of oblivion of central serial items, which we gave the epithet of “middlecy”, is a further example of the complex relationship between emotion and memory, not limited to primacy and recency phenomena.

## Data Availability

Data are available from the corresponding author on request.

## Appendix A

**Table A1:** Mean (range) of valence and arousal rating values for each block and experimental condition.

			Block 1	Block 2	Block 3	Block 4	Block 5
**Negative context condition**	Negative images	*Valence*	5.3 (0.7–10.5)	5.7 (0.8–10.8)	6.2 (1.8–10.9)	6.8 (1.9–11.8)	7.3 (2.0–12.8)
		*Arousal*	77.2 (60.5–92.4)	78.4 (68.5–91.3)	75.3 (68.8–83.4)	78.5 (75.2–83.9)	77.2 (70.6–87.3)
	Neutral images	*Valence*	57.0 (49.1–62.2)	60.8 (54.1–68.9)	54.4 (43.1–67.0)	57.9 (49.4–67.8)	54.5 (45.2–65.7)
		*Arousal*	24.2 (10.2–41.5)	23.8 (14.3–29.8)	21.5 (16.5–26.8)	24.2 (17.9–33.4)	23.5 (10.7–43.1)
**Positive context condition**	Positive images	*Valence*	96.2 (92.9–98.7)	96.2 (94.8–97.8)	96.4 (95.2–97.8)	96.5 (95.2–98.2)	96.6 (95.3–98.3)
		*Arousal*	19.9 (8.0–35.8)	21.8 (10.4–34.7)	17.9 (5.9–29.3)	17.1 (8.5–22.5)	12.2 (11.4–14.7)
	Neutral images	*Valence*	55.4 (44.5–67.7)	55.6 (48.9–62.6)	54.6 (49.7–59.8)	53.5 (46.4–61.8)	53.9 (41.0–62.8)
		*Arousal*	29.1 (17.7–43.8)	26.8 (13.3–37.6)	25.2 (12.1–43.1)	23.8 (13.7–32.8)	27.6 (18.3–40.4)

Values in the valence scale ranged from 0 = ‘very negative picture’ to 100 = ‘very positive picture’, with 50 = ‘neutral’. Values in the arousal scale ranged from 0 = ‘calm’ to 100 = ‘excited’. Values for each image were taken by Dan-Glauser and Scherer 2011.

**Table A2:** Lists of words used in the experiments (Italian in brackets).

	**List 1**	**List 2**	**List 3**	**List 4**	**List 5**
1	Patch (Pezza)	Year (Anno)	Hole (Buco)	Fork (Forchetta)	Future (Futuro)
2	Pole (Palo)	Wall (Muro)	Tongue (Lingua)	Drum (Tamburo)	Cream (Pomata)
3	Mount (Monte)	Stone (Pietra)	Blackbird (Merlo)	Pigeon (Piccione)	Smoke (Fumata)
4	Dog (Cane)	Ship (Nave)	Bag (Borsa)	Fort (Fortino)	Clean (Lavato)
5	Shore (Riva)	Pizza (Pizza)	World (Mondo)	Final (Finale)	Carpet (Tappeto)
6	Night (Notte)	Blackberry (Mora)	Horn (Corno)	Carrot (Carota)	Drink (Bibita)
7	Walnut (Noce)	Heart (Cuore)	Mouse (Topo)	Table (Tavolo)	Donkey (Somaro)
8	Paper (Carta)	Paw (Zampa)	Barrel (Botte)	Size (Misura)	Furniture (Mobile)
9	Trot (Trotto)	Name (Nome)	Door (Porta)	Pan (Padella)	Pistachio (Pistacchio)
10	Chicken (Pollo)	Little (Poco)	Crest (Cresta)	Boar (Cinghiale)	Pudding (Budino)
11	Oil (Olio)	Pipe (Pipa)	Bull (Toro)	Racing car (Bolide)	Meatball (Polpetta)
12	Thrush (Tordo)	Leaf (Foglia)	Beautiful (Bello)	Steam (Vapore)	Vanity (Vanità)
13	Pumpkin (Zucca)	Woman (Donna)	Backpack (Zaino)	Sister (Sorella)	Moral (Morale)
14	Tree (Albero)	Tuna (Tonno)	Sky (Cielo)	Mattino (Mattino)	Camel (Cammello)
15	Orange (Arancio)	Mouth (Bocca)	Glass (Vetro)	Cabbage (Cavolo)	Cicada (Cicala)
	**List 6**	**List 7**	**List 8**	**List 9**	**List 10**
1	Hedgehog (Riccio)	Rice (Riso)	Drop (Goccia)	Bristle (Setola)	Horse (Cavallo)
2	Grass (Erba)	Peach (Pesca)	Curtain (Tenda)	Sweater (Maglione)	Palate (Palato)
3	City (Città)	Key (Chiave)	Wheel (Ruota)	Smile (Sorriso)	News (Novità)
4	Rhyme (Rima)	Dice (Dado)	Dream (Sogno)	Rubber dinghy (Canotto)	Channel (Canale)
5	Doubt (Dubbio)	Line (Riga)	Marble (Marmo)	Millstone (Macina)	Shovel (Badile)
6	Song (Canto)	Boat (Barca)	Turkish (Turco)	Barrel (Barile)	Swamp (Palude)
7	Beak (Becco)	Pocket (Tasca)	Lips (Labbra)	Shrimp (Gambero)	Finished (Finito)
8	Goat (Capra)	Voice (Voce)	Money (Soldo)	Cinema (Cinema)	Nomad (Nomade)
9	Thorn (Spina)	Net (Netto)	Cork (Tappo)	Monday (Lunedì)	Kangaroo (Canguro)
10	Broken (Rotto)	School desk (Banco)	Thief (Ladro)	Box (Scatola)	Hammer (Martello)
11	Rock (Sasso)	Canvas (Tela)	Tin (Latta)	Steak (Bistecca)	Bin (Bidone)
12	Frog (Rana)	Stain (Macchia)	Worm (Verme)	Comb (Pettine)	Hair (Capelli)
13	Roller (Rullo)	Bed (Letto)	Much (Tanto)	Cloud (Nuvola)	Famous (Famoso)
14	Man (Uomo)	Jar (Vaso)	Jump (Salto)	Tomorrow (Domani)	Goalkeeper (Portiere)
15	Pen (Biro)	House (Casa)	Sign (Segno)	Building (Palazzo)	Thimble (Ditale)
	**List 11**	**List 12**	**List 13**	**List 14**	**List 15**
1	Saw (Sega)	Lot (Lotto)	Cart (Carretto)	Husband (Marito)	Parade (Parata)
2	Bread (Pane)	Butter (Burro)	Number (Numero)	Fable (Favola)	Tender (Tenero)
3	Day (Giorno)	Fish (Pesce)	Faith (Fede)	Buffalo (Bufalo)	Venal (Venale)
4	End (Fine)	Crippled (Zoppo)	Rotated (Ruotato)	Muffler (Marmitta)	Flag (Bandiera)
5	Cake (Torta)	Tube (Tubo)	Salami (Salame)	Duck (Anatra)	Ill (Malato)
6	Lard (Lardo)	Fear (Paura)	Knob (Pomello)	Banal (Banale)	Epiphany (Befana)
7	Kick (Tiro)	Kidney (Rene)	Bottle (Bottiglia)	Ship wheel (Timone)	Brick (Mattone)
8	Hay (Fieno)	White (Bianco)	Abito (Dress)	Wound (Ferita)	Insect (Insetto)
9	Lyre (Lira)	Straight (Dritto)	Boiled (Bollito)	Dinette (Tinello)	Dolphin (Delfino)
10	Face (Viso)	Branch (Ramo)	Work (Lavoro)	Eel (Anguilla)	Cliff (Dirupo)
11	Air (Aria)	Hard (Duro)	Folder (Cartella)	Candle (Candela)	Winged (Alato)
12	Mule (Mulo)	Well (Pozzo)	Appointment (Nomina)	Abbot (Abate)	Ball (Pallone)
13	Good (Bene)	Sheet (Foglio)	Giraffe (Giraffa)	Alien (Alieno)	Total (Totale)
14	Makeup (Trucco)	Brawl (Rissa)	Truth (Verità)	Storm (Tempesta)	Pheasant (Fagiano)
15	Moon (Luna)	Quill (Penna)	Sandwich (Panino)	Praise (Lodato)	Flour (Farina)
